# Clinical Genetics in Interstitial Lung Disease

**DOI:** 10.3389/fmed.2018.00116

**Published:** 2018-04-26

**Authors:** Chad A. Newton, Philip L. Molyneaux, Justin M. Oldham

**Affiliations:** ^1^Eugene McDermott Centre for Human Growth and Development, University of Texas Southwestern Medical Center at Dallas, Dallas, TX, United States; ^2^Division of Pulmonary and Critical Care Medicine, Department of Medicine, University of Texas Southwestern Medical Center, Dallas, TX, United States; ^3^Fibrosis Research Group, National Heart and Lung Institute, Imperial College, London, United Kingdom; ^4^National Institute for Health Research Respiratory Biomedical Research Unit, Royal Brompton Hospital, London, United Kingdom; ^5^Division of Pulmonary, Department of Medicine, Critical Care and Sleep Medicine, University of California at Davis, Davis, CA, United States

**Keywords:** idiopathic pulmonary fibrosis, hypersensitivity pneumonitis, interstitial lung disease, idiopathic interstitial pneumonia, genomics

## Abstract

Interstitial lung disease (ILD) comprises a heterogeneous group of diffuse parenchymal lung processes with overlapping clinical, radiographic, and histopathologic features. Among the most common and deadly ILDs are idiopathic pulmonary fibrosis (IPF) and chronic hypersensitivity pneumonitis (CHP). As the name implies, the cause of IPF remains elusive, but a variety of genetic and infectious risk factors have been identified. CHP results from chronic inhalation of an organic antigen, usually of avian or mold origin, and may occur in patients with a genetic predisposition. While IPF is treated with anti-fibrotic compounds, CHP is generally treated by suppression of the immune system and elimination of the causative antigen. Despite advances in our understanding of IPF and CHP, there exists substantial variability in the diagnosis and treatment of these disease processes. Furthermore, IPF and CHP natural history and treatment response remain far from uniform, leaving it unclear which patients derive the most benefit from disease-specific therapy. While clinical prediction models have improved our understanding of outcome risk in patients with various forms of ILD, recent advances in genomic technology provides a valuable opportunity to begin understanding the basis for outcome variability. Such advances will ultimately allow for the incorporation of genomic markers into risk stratification and clinical decision-making. In this piece, we highlight recent advances in our understanding of the genomic factors that influence susceptibility and outcome risk among patients with IPF and CHP. Genomic modalities used to identify these genomic markers include genome-wide association studies, analyses of gene expression, drug–gene interaction testing, telomere length determination, telomerase mutation analysis, and studies of the lung microbiome. We then identify gaps in knowledge that should be addressed to help facilitate the incorporation of these genomic technologies into ILD clinical practice.

## Introduction

The interstitial lung diseases (ILDs) are comprised of a heterogeneous group of diffuse parenchymal lung processes with overlapping clinical, radiographic, and histopathologic features (1). Among the most common ILDs are idiopathic pulmonary fibrosis (IPF) and chronic hypersensitivity pneumonitis (CHP). IPF is progressive fibrosing interstitial pneumonia of unclear etiology with recently identified genetic and microbial risk factors (2–7). CHP results from an inappropriate immunologic response to chronic inhalation of organic antigen, usually of avian or mold origin, and results in pulmonary fibrosis after prolonged exposure (8). Like IPF, one’s genetic makeup likely influences CHP susceptibility (9–11).

Idiopathic pulmonary fibrosis is now treated with compounds targeting fibrotic mediators after phase III clinical trials demonstrated efficacy in slowing pulmonary function decline (12–14). Prospective treatment data for CHP is lacking, but management is generally geared toward attempted removal of the causative antigen, along with variable suppression of the immune system, as this has been associated with stability in pulmonary function (15, 16). While these disease-specific treatment approaches appear to favorably impact disease course, there remains substantial variability in outcomes within IPF and CHP. Recent advances in genomic technologies have provided a valuable opportunity to begin understanding the basis for this outcome variability.

In this review, we highlight recently identified genomic factors influencing susceptibility and outcomes of patients with IPF and CHP. These include single-nucleotide polymorphisms (SNPs) identified by targeted sequencing and genome-wide association studies (GWAS), gene expression profiling, telomere length testing, and lung microbiome bacterial DNA profiling. We then identify gaps in knowledge that should be addressed to help facilitate the incorporation of these genomic technologies into ILD clinical practice.

### Gene Polymorphisms

Three GWAS have been performed in patients with IPF to date, which identified SNPs within a number of loci to be associated with IPF susceptibility (2, 3, 6). Among the variants identified by this approach were several on the short arm of chromosome 11, including a SNP in the promoter of *MUC5B* (rs35705950) and an intronic SNP near *TOLLIP* (rs5743890)*. MUC5B* encodes one of several mucin-producing genes, which facilitate airway clearance and function to maintain immune homeostasis (5, 17, 18). *TOLLIP* encodes toll-interacting protein, which inhibits toll-like receptor signaling and acts as a critical mediator of airway host defense (17, 19–22). The *MUC5B* promoter SNP increases the risk of developing IPF by roughly threefold, while the intronic *TOLLIP* SNP reduces the risk by about 70%. The *MUC5B* promoter polymorphism has also been shown to increase the risk of developing interstitial lung abnormalities (ILA) among the general population (23). While the proportion of patients with an ILA that ultimately develop IPF remains unknown, this variant does appear to increase risk of progressive disease (24).

**Figure 1:**
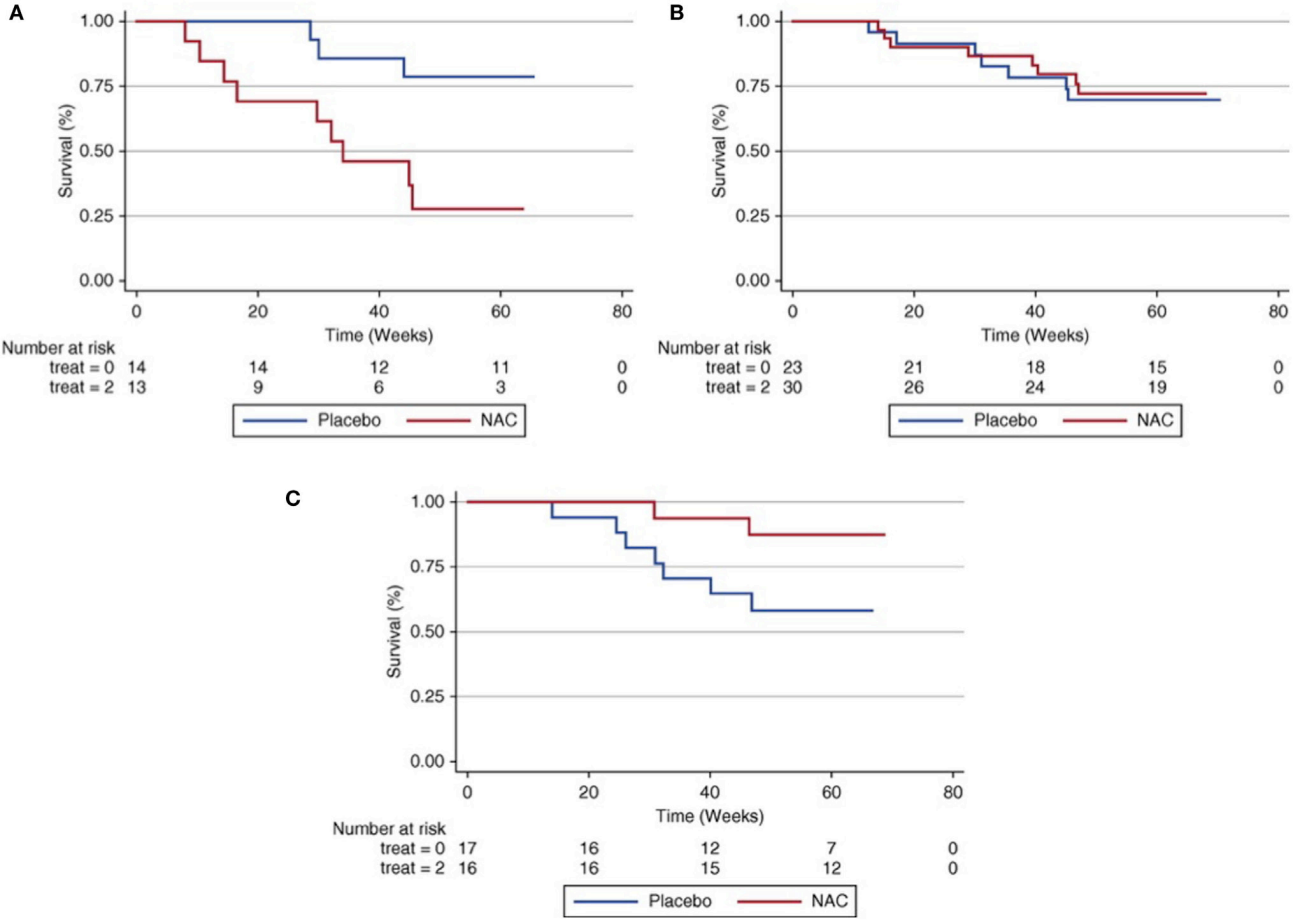
Composite endpoint-free survival between N-acetylcysteine (NAC) and placebo groups after stratification by rs3750920 (TOLLIP) genotype. In those with a CC genotype **(A)**, NAC therapy is associated with worse survival than placebo [*P*_logrank_ = 0.01; hazard ratio (HR), 3.23; 95% confidence interval (CI), 0.79–13.16; *P* = 0.10]. In those with a CT genotype **(B)**, survival is similar between groups (*P*_logrank_ = 0.82; HR 0.76; 95% CI 0.27–2.19; *P* = 0.62). In those with a TT genotype **(C)**, NAC therapy is associated with improved survival compared with placebo (*P*_logrank_ = 0.06; HR 0.14; 95% CI 0.02–0.83; *P* = 0.03). Multivariable Cox regression models adjusted for age, sex, forced vital capacity (percentage predicted), and diffusion capacity of the lung for carbon monoxide (percentage predicted) at time of study enrollment. Reprinted from Ref. (28) with permission of the American Thoracic Society. Copyright © 2016 American Thoracic Society.

Besides influencing IPF susceptibility, SNPs within *MUC5B* and *TOLLIP* may also have prognostic significance. While increasing IPF susceptibility risk, the *MUC5B* promoter SNP is paradoxically associated with a twofold decrease in mortality risk (25). A similar finding is observed with the intronic *TOLLIP* SNP, which is associated with a 65% increase in mortality risk, despite reducing the risk of developing IPF (6). A recent pharmacogenetic investigation sought to determine whether relevant variants in *TOLLIP* and *MUC5B* may influence IPF treatment response. Using paired clinical and genotype data from patients enrolled in the previously completed effectiveness of Prednisone, Azathioprine, and N-Acetylcysteine in Patients with Idiopathic Pulmonary Fibrosis (PANTHER) trial (26, 27), investigators showed that an exonic SNP within *TOLLIP* (rs3750920) was associated with a favorable responsive to N-acetylcysteine (NAC) (28). Compared to placebo, those with the TT genotype at this SNP treated with NAC had a significantly reduced composite endpoint risk, including death, hospitalization, and forced vital capacity decline (Figure 1). Those with the CC genotype treated with NAC had a trend toward harm when compared to placebo and outcomes were similar in those with the CT genotype. Approximately 25% of patients with IPF carry both copies of this polymorphism, suggesting that NAC may benefit a large minority of IPF patients if these findings are replicated in a prospective clinical trial.

No GWAS have been performed in patients with CHP to date, but studies employing targeted genotyping have identified gene variants linked to disease susceptibility and outcomes. Camarena and colleagues conducted targeted genotyping of SNPs within the major histocompatibility complex (MHC) II region and found that SNPs within HLA-DRB1 were disproportionately present in patients with avian antigen-associated CHP compared to control subjects (11). Subsequent studies by this group implicated polymorphisms in transporter-associated antigen processing genes and tumor necrosis factor as potential risk factors for CHP susceptibility (29). Ley and colleagues recently showed the MUC5B promoter polymorphism linked to IPF susceptibility to be present in a significantly higher proportion of patients with CHP compared to healthy controls (30). However, as opposed to patients with IPF, the MUC5B promoter SNP was associated with increased mortality risk in those with CHP, though the strength of this association varied across cohorts. These investigators also assessed the intronic TOLLIP SNP previously linked to IPF, but found no association with either susceptibility or survival in those with CHP.

### Gene Expression

While GWAS have identified gene polymorphisms that may influence IPF susceptibility, transcriptomic analyses of RNA isolated from lung tissue and peripheral blood have shed important light on gene expression pathways involved in IPF and CHP pathogenesis and outcomes. Selman and colleagues conducted a microarray analysis of RNA obtained from lung tissue in patients with IPF and CHP to determine whether gene expression profiles could differentiate these disease processes. These investigators showed that while patients with IPF had upregulation of genes involved in tissue remodeling, apoptosis and fibroblast signaling, those with CHP displayed upregulation of genes critical to immunologic function, including those T cell signaling and others related to MHC function (9).

Subsequent transcriptomic investigations using lung and peripheral blood specimens from patients with IPF supported the role of genes involved in alveolar epithelial injury and remodeling in IPF pathogenesis (31, 32). Yang and colleagues showed that alpha defensin signaling in the peripheral blood may play a role in disease progression, as differential expression of this and other associated pathways characterized disease severity in these patients (32). Selman and colleagues showed that compared to IPF patients with relatively stable disease, lung tissue of patients with accelerated disease progression displayed an overexpression of genes involved in oxidative stress and fibroblast proliferation (31). These data suggest that unique molecular phenotypes exist that may help better predict disease trajectory.

**Figure 2:**
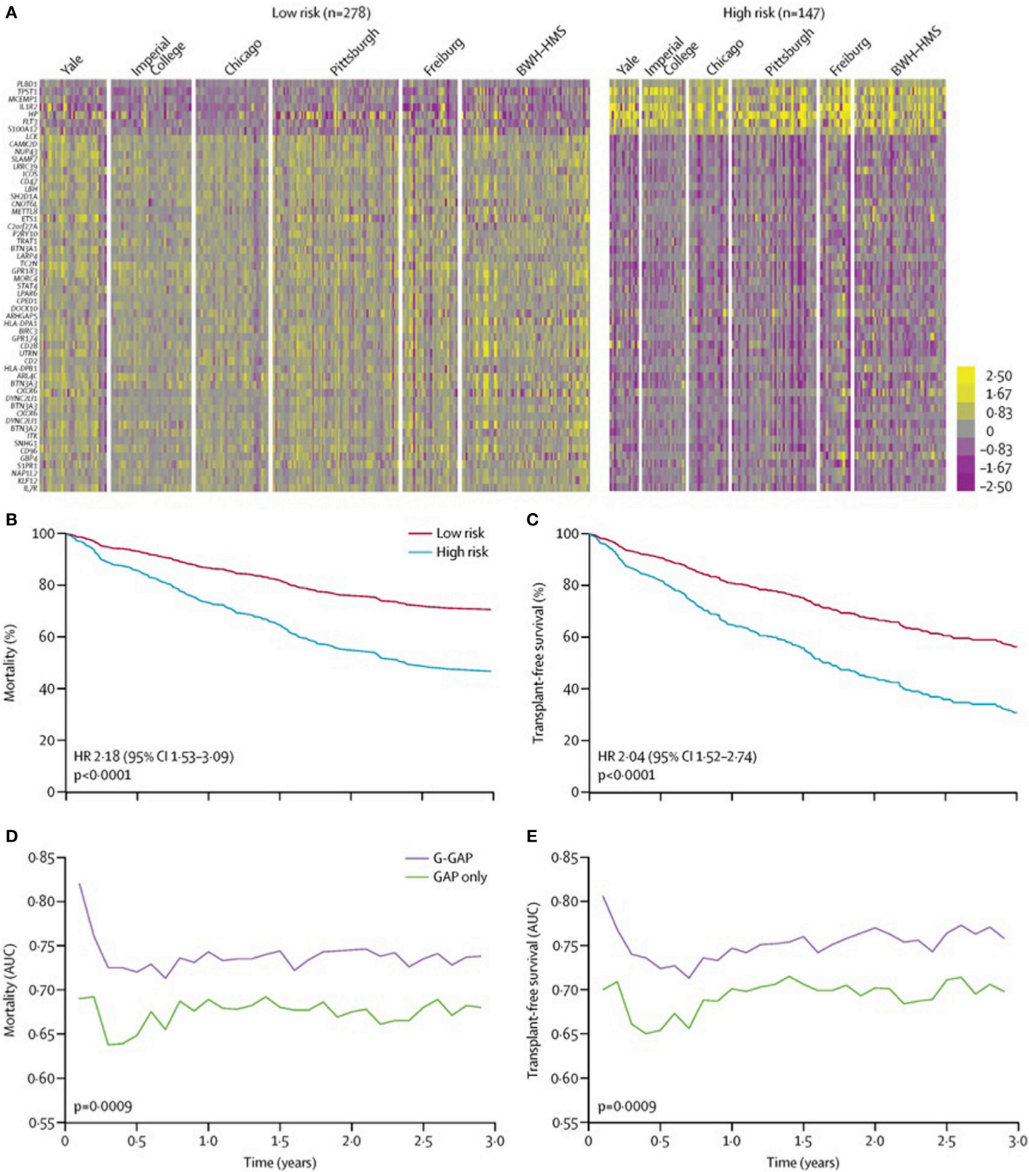
52-gene risk profiles and outcomes independent of demographic and clinical variables. **(A)** Pooled data analysis comparing high-risk profile patients with low-risk profile patients from all cohorts. Color scale is shown adjacent to heat maps in log-based two scale. Mortality **(B)** and transplant-free survival **(C)** differ between high-risk and low-risk patients from all cohorts after adjusting for age, sex, FVC%, and immunosuppressive therapy. AUC of time-dependent receiver operating characteristic analysis for mortality **(D)** and transplant-free survival **(E)** based on the GAP index alone or the G-GAP index in all patients. Abbreviations: BWH–HMS, Brigham and Women’s Hospital–Harvard Medical School; FVC, forced vital capacity; AUC, area under the curve; GAP, Gender, Age, and Physiology; G-GAP, GAP and genomic; HR, hazard ratio. Reprinted from Ref. (35) with permission from Elsevier. Copyright © 2017 Elsevier.

In addition to differentiating IPF from other forms of ILD, investigators have utilized transcriptomic analysis to develop a peripheral blood-based genomic prediction tool to predict IPF survival. Using a two-stage, multi-center derivation and validation approach, Herazo-Maya and colleagues identified a gene signature composed of 52 differentially expressed genes could effectively categorize patients with high versus low mortality risk over a 4-year follow-up period (33). This gene signature had similar test performance characteristics as a validated clinical prediction model (34) and significantly improved the clinical model when the gene signature was incorporated. These investigators then validated this 52-gene signature across 6 centers in the United States and Europe (Figure 2) and showed that initiation of anti-fibrotic therapy was associated with favorable modulation of the gene signature (35). Many of the differentially expressed genes identified by this approach are critical to immunologic activation, suggesting that dysregulation of the immune response may contribute to IPF progression.

### Telomere Length

Studying large families with multiple affected members led to the discovery of multiple genes associated with monogenetic forms of familial pulmonary fibrosis (FPF) and improved our understanding of the genetic underpinnings of ILD. To date, there have been seven telomere-related genes that have been implicated in adult-onset FPF (*TERT, TERC, RTEL1, PARN, NAF1, TINF2, DKC1*) (36–43). Pathogenic variants in telomere-related genes are associated with extremely short age-adjusted telomere length that predispose to multisystem organ dysfunction, including pulmonary fibrosis, liver dysfunction, and bone marrow failure (44, 45).

Telomeres, or the ends of chromosomes, solve the end replication problem and prevent the activation of DNA damage pathways. Telomere-related pathogenic variants are found in ~30% of all FPF kindred (36, 37, 46–48); *TERT* is the most commonly affected gene and accounts for ~20% of FPF (36, 38). The inheritance of a telomere-related pathogenic variant confers substantial risk for ILD development; however, other factors such as age, gender, environmental exposures, and telomere length also contribute to the variability in penetrance (36, 46–48). Unfortunately, there is poor genotype-ILD phenotype correlation in individuals with telomere-related pathogenic variants. While IPF is the most common clinical diagnosis among these FPF kindred, it accounts for less than half of cases; the other portion of FPF includes ILD of both known (CHP and connective tissue disease-associated ILD) and unknown causes (idiopathic nonspecific interstitial pneumonia and idiopathic pleuroparenchymal fibroelastosis) (48). Interestingly, the presence of a telomere-related rare variant in *TERT, TERC, PARN*, or *RTEL1* is associated with rapid disease progression and poor survival regardless of the diagnosis (48). This finding implies that the presence of a pathogenic variant in a telomere-related gene trumps the clinical diagnosis in terms of disease behavior and overall prognosis. This also suggests that telomere dysfunction not only predisposes to disease development but may also be involved in disease progression and fibrosis propagation.

**Figure 3:**
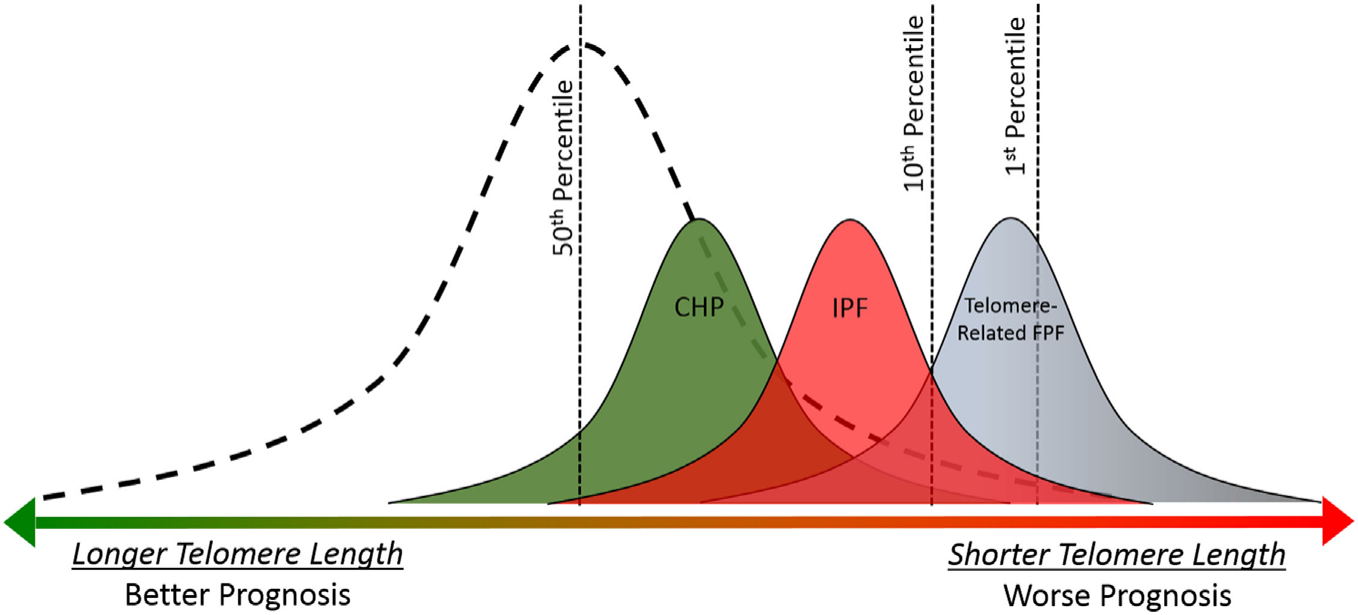
Telomere length is associated with prognosis in idiopathic pulmonary fibrosis (IPF), chronic hypersensitivity pneumonitis (CHP), and telomere-related familial pulmonary fibrosis (FPF). Telomere lengths for healthy controls follow a normal distribution (dashed line). Mean telomere length for CHP, IPF, and telomere-related FPF cohorts are shorter than healthy controls (49, 50). Overall prognosis and mean telomere length follow similar pattern across diagnoses (CHP > IPF > telomere-related FPF); and shorter individual telomere length is associated with worse prognosis in patients with IPF and CHP (49, 30). The presence of a rare variant in the telomere-related genes (*TERT, TERC, PARN*, or *RTEL1*) is associated with extremely short telomere length and poor prognosis (37, 48).

Short age-adjusted telomere lengths are found more commonly in ILD patients than rare genetic variants (46) and are present across a wide variety of ILDs, including IPF and CHP (49, 50). Short telomere length is relatively common in both of these diseases, 23–50% of patients with sporadic IPF and 24% of CHP patients have age-adjusted telomere length less than 10th percentile (46, 51, 30). Similar to telomere-related rare variants, the presence of short telomere length is associated with poor prognosis in patients with IPF and CHP (Figure 3).

The association between short telomere length and survival in IPF has been replicated in multiple independent cohorts (49, 52), and recently this association was expanded to patients with CHP (30). There is significant overlap between the clinical, radiographic, and histopathologic features of IPF and CHP. Telomere length may be partly responsible for this overlap since short age-adjusted telomere length is associated with radiographic and histopathologic “IPF-like features” including honeycombing, temporal heterogeneity, and fibroblastic foci in patients with well characterized CHP (30). Further studies are needed across other subtypes of ILD, such as autoimmune-mediated ILD, to determine if short telomere length represents a robust predictor of prognosis or disease progression across clinical diagnoses. If so, this would argue that molecular classification, specifically with telomere length, could improve our ability to predict disease course in a wide variety of ILD subtypes.

### Lung Microbiome

Genetic susceptibility alone is not enough to develop pulmonary fibrosis and an environmental trigger is likely required to initiate the fibrotic cascade. Many environmental factors associated with IPF susceptibility have been identified, but historically most research of infective agents has focused on the role of viruses in the pathogenesis and progression of IPF (53). This was in part due to the incorrectly held mantra that the lungs were sterile outside of times of clinical infection and also due to the limited tools available in our armamentarium; almost 70% of mucosal bacteria cannot be cultured (54).

Molecular, culture independent, microbiology has benefited from the explosion of sequencing technologies in the past decade, which have transformed the microbial ecology landscape. High throughput large-scale studies relying on genetic identification of the bacterial housekeeping 16S-rRNA gene can now identify bacterial species that were previously unable to be cultured. The epithelial surfaces of the respiratory tract, previously thought to be sterile, have been shown using these culture-independent techniques to accommodate dynamic microbial communities in health and disease (55). These communities are surprisingly stable in healthy individuals (55). In disease, this normal harmony is disrupted with distinct bacterial communities seen in asthma, COPD, bronchiectasis, and cystic fibrosis (55–57). In recent years, we have come to understand these communities are also altered in pulmonary fibrosis (58, 59).

The first application of a culture-independent molecular technique in ILD studied the microbiome in bronchoalveolar lavage (BAL) from patients diagnosed with a variety of idiopathic interstitial pneumonias using 16S-rRNA gene PCR and degenerating gel electrophoresis (60). This was followed by a study investigating the upper and lower respiratory tract microbiota in a group of patients with ILD compared to healthy controls (61). These initial studies demonstrated the presence of bacterial DNA in the lower airways of patients with ILD, but revealed no significant differences in the microbiome between these patients and healthy controls. The first study to employ these techniques to study the microbiome in fibrotic lung disease on a large scale was undertaken as part of the Correlating Outcomes with biochemical Markers to Estimate Time-progression in IPF (COMET) study (4). A subset of individuals enrolled in COMET underwent BAL at time of enrollment, which was analyzed for association between microbiome indices and disease outcomes. Investigators identified an association between disease progression and the relative abundance of two specific *Steptococcus* and *Staphylococcus* OTUs. By dichotomizing patients into cohorts with high and low numbers of these bacterial OTUs, these authors demonstrated clear differences in survival. Despite this observation, however, few patients had bacterial levels above the statistical significance threshold, suggesting they alone did not explain disease progression (62). The retrospective nature of this investigation, along with lack of control subjects, limited the conclusions that could be drawn.

**Figure 4:**
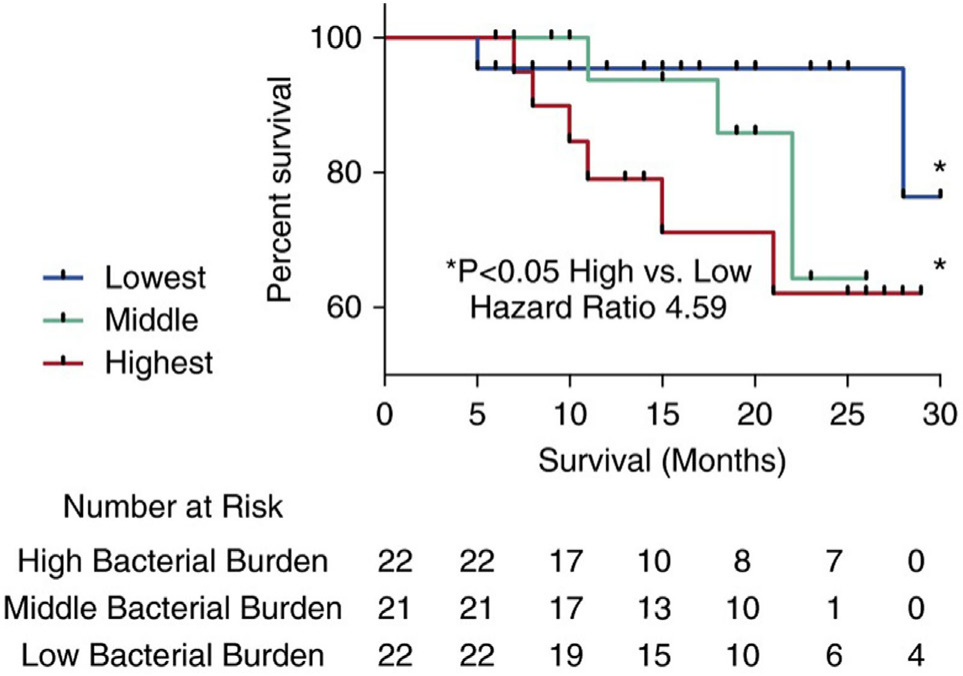
Kaplan–Meier curves for time until death. Subjects with idiopathic pulmonary fibrosis (IPF) in the top tertile with the highest bacterial load (16S copy number per mL of bronchoalveolar lavage) (depicted by the large dashed line) were at increased risk of mortality compared to IPF subjects in the tertile with the lowest bacterial burden (depicted by a solid line) (hazard ratio 4.59) (95% confidence interval, 1.05–20). Reprinted from Ref. (5) with permission of the American Thoracic Society. Copyright © 2016 American Thoracic Society.

A subsequent prospective study of the lung microbiome compared IPF cases to healthy control subjects and controls with COPD, allowing for direct compassions between health and disease (5). This investigation demonstrated higher numbers of *Veillonella, Neisseria, Streptococcus*, and *Haemophilus* spp. in patients with IPF compared to controls. The most striking differences, however, were observed in the bacterial burden, which was increased twofold in IPF patients compared to control subjects. Within the IPF cohort, bacterial burden correlated with disease progression. When stratifying the cohort by this metric, the authors demonstrated a clear increase in mortality risk with increasing bacterial burden (Figure 4).

These authors also showed few differences in the microbiome between IPF subjects with progressive or stable disease, suggesting bacterial load itself might be more important in driving disease progression. The authors hypothesized a mechanistic link between host and environment and demonstrated an association with bacterial burden and the *MUC5B* promoter polymorphism, with individuals carrying the minor allele of this SNP having a lower bacterial burden. Driven by these tantalizing interactions between host and environment, authors of both microbiome studies have attempted to advance the studies of the lung microbiome from merely descriptive and observational to functional (63, 64). Integrating microbial data with peripheral blood transcriptome data demonstrates an association between the microbiome and upregulation of genes involved in host defense and bacterial clearance. Indeed, in subjects in the COMET study patients with IPF and a downregulated peripheral immune response had higher bacterial loads of *Streptococcus* and *Pseudomonas* and worse survival.

## Gaps in Knowledge and Potential Clinical Applications

While some SNPs associated with IPF susceptibility, notably those within *MUC5B* and *TOLLIP*, influence both susceptibility and mortality risk, few others have demonstrated significant outcome association (6, 28). Given the substantial heterogeneity in IPF natural history (31, 65), it stands to reason that genomic factors influencing IPF susceptibility may be independent of those influencing IPF survival. The aforementioned GWAS were designed to identify SNPs disproportionately present in patients with IPF relative to healthy controls. A case-only GWAS specifically designed to identify SNPs linked to IPF survival has the potential to identify novel genes involved in IPF progression and may improve upon current outcome prediction models for patients with IPF and other forms of fibrotic ILD (34, 66).

The development of a transcriptomic signature to predict mortality has greatly improved our understanding of IPF pathobiology. Now that IPF has two currently approved therapies, it will be important to assess the test performance characteristics of this signature in those treated with prolonged anti-fibrotic therapy and in those without prior exposure to immunosuppressive therapy, as was common practice prior to completion of the PANTHER trial (27). In addition, the use of this gene signature to predict biologic responsiveness to anti-fibrotic therapy holds great promise as the field moves toward an era of personalized medicine. While mortality remains the most important endpoint for patients and clinicians alike, the development of additional transcriptomic signatures to predict other clinically relevant endpoints, such as pulmonary function decline, has the potential to guide clinical trial design through enrichment of clinical trial cohorts with patients at high risk for meeting the trial primary endpoint.

The presence of telomere-related rare variants or short telomere length predispose to rapid disease progression in both IPF and CHP, however, there is very little data regarding response to specific treatments. The therapeutic strategies for IPF and CHP differ substantially. Immune suppression is often employed for patients with CHP and progressive disease, while immunosuppression is detrimental in IPF (27). To our knowledge, the safety or efficacy of immunosuppression in patients with short telomeres and ILD has not been systematically tested. However, small case series of patients with rare variants in *TERT* and *TERC* suggest that immunosuppression after lung transplant for ILD may be associated with high rates of side effects including bone marrow failure, liver toxicity, and infections (67–69). This raises the question of safety and tolerability of this therapeutic strategy for patients with short telomere length across a wide variety of ILDs that are often treated with immune suppression. Anti-fibrotic medications, including pirfenidone and nintedanib, are effective at slowing lung function decline in patients with IPF (12–14), but their effectiveness in CHP is unknown. Pirfenidone was well tolerated in a small cohort of *TERT* carriers (70) but larger studies are needed to determine efficacy in patients with telomere dysfunction.

Idiopathic pulmonary fibrosis is characterized by a distinct respiratory microbiome, with a higher bacterial burden than in health. This is further disturbed during exacerbations of disease (71). Despite advances in our understanding of how the microbiome may influence disease susceptibility and progression, a causal, mechanistic link to these observations has yet to be delineated. Additionally a number of technical challenges remain for studies of the lung microbiome, and future work will need to address these (72, 73). The role of the microbiome in treatment response also remains unclearly defined. Two clinical trials assessing the efficacy of antibiotic therapy for patients with IPF—The Efficacy and Mechanism Evaluation of Treating Idiopathic Pulmonary fibrosis with the Addition of Co-trimoxazole (ISRCTN17464641) and Study of Clinical Efficacy of Antimicrobial Therapy Strategy Using Pragmatic Design in Idiopathic Pulmonary Fibrosis (CleanUp-IPF) (NCT02759120)—are currently enrolling. These trials will not only assess how co-trimoxazole (or doxycycline) therapy impact relevant IPF outcomes but also will provide the opportunity to study how the lung microbiome may be altered by these therapies.

The majority of genomic data generated to date has been in patients with IPF, leaving CHP ripe for similar investigation. However, standardization of diagnostic criteria through international consensus is first needed. Once that occurs, GWAS to identify SNPs linked to disease susceptibility and survival would advance our understanding of disease underpinnings and potentially identify novel therapeutic targets for this devastating disease without proven therapy. In addition, determining whether outcome-related transcriptomic signatures derived in patients with IPF informs outcomes in patients with CHP will be of immense clinical value. Finally, determining whether the microbiota makeup of patients with CHP influences disease susceptibility and outcomes has the potential to guide therapy in these patients.

## Conclusion

In this review, we highlight the most developed genomic technologies informing susceptibility and outcome risk in patients with IPF and CHP. There remain critical questions to be answered to characterize the extent to which these technologies will improve risk stratification. In addition, a cost benefit analysis will be necessary to determine whether individual technologies make sense from a cost utilization perspective. The field of ILD has advanced rapidly over the last 10 years and will continue to do so into the foreseeable future. Clinical genetics represents the logical next step for the field and holds great potential to be a cornerstone of personalized medicine in the field.

## Author Contributions

CN, PM, and JO contributed to the conception and writing of this review. All authors have reviewed and approved the submitted work.

## Conflict of Interest Statement

CN, PM, and JO have no relevant conflicts to disclose related to the submitted work.
